# Beer Potomania: Why Initial Fluid Resuscitation May Be Harmful

**DOI:** 10.1155/2022/8778304

**Published:** 2022-04-22

**Authors:** Zhuo Lin Yu, Lisa Fisher

**Affiliations:** ^1^Stony Brook University Hospital, 101 Nicolls Rd, Stony Brook, NY 11794, USA; ^2^Northport VA Medical Center, 79 Middleville Rd, Northport, NY 11768, USA

## Abstract

Beer potomania is one of the less common causes of hyponatremia that we encounter. Patients usually have a recent history of binge drinking along with poor diet. The low solute content in alcoholic beverages limits daily urine output, and ingestion of extra fluid will cause dilutional hyponatremia as a result. Blindly providing intravenous fluid without an underlying cause of the hyponatremia can be detrimental, such as in patients with beer potomania. In our case, a patient presented to the emergency department due to poor oral intake from jaw pain and was found to be hyponatremic from alcohol intake. He initially received 2 liters of fluid, which caused overcorrection of his sodium, requiring more free water to lower his sodium as a result.

## 1. Background

Our case occurs at a university hospital in Long Island, New York.

## 2. Objective

Our case discusses the underlying pathophysiology of beer potomania, and the reason that beer potomania patients are prone to overcorrection of sodium levels with intravenous fluid infusion.

### 2.1. Patient Information

The patient is a 53-year-old man with a past medical history of metastatic squamous cell carcinoma of the head and neck treated with laser ablation and multiple oral surgeries in the past, alcohol abuse, and nicotine dependence. He presented to the hospital due to worsening jaw pain and decreased oral intake. The patient reported feeling fatigued but had no fever or chills. He was not taking any medications at home. Due to jaw pain, he also had not been eating much, but instead had been drinking two six-packs of beer and one pint of vodka daily.

### 2.2. Physical Exam

On presentation, he was afebrile with a temperature of 36.9 C, heart rate 89, blood pressure 92/60, and saturated 95% on room air. On physical exam, he appeared thin and cachectic but comfortable without signs of distress. He had bitemporal wasting, dry mucous membrane, and a large irregular mass extending from the lower jaw to the submandibular region. His cardiac and pulmonary exams were unremarkable. There was no focal neurologic deficit observed.

### 2.3. Diagnostic Assessment

Initial laboratory tests showed the following: WBC 10.02 k/*μ*L, RBC 4.18 k/*μ*L, hemoglobin 13.3 g/dL, hematocrit 39%, sodium 113 mmol/L, chloride 70 mmol/L, potassium 4 mmol/L, CO_2_ 34 mmol/L (27 mmol/L 4 hours later and stayed within normal range on subsequent labs), AG 9, BUN 8 mg/dL, Cr 0.43 mg/dL, magnesium 1.4 mg/dL, ALT 19 IU/L, AST 44 IU/L, alkaline phosphate 152 IU/L, and albumin 3.2 g/dL. The rest of the biochemical analysis including calcium, phosphorus, and glucose were within normal range.

Given the patient's low serum sodium, we obtained more labs for further workup of his hyponatremia. Further tests showed plasma osmolality 258 mOsm/kg (275–300 mOsm/kg), sodium in the urine sample was <15 mmol/L, and urine osmolality 94 mOsm/kg. On urine analysis, the sample was negative for nitrite or leukocyte esterase, but specific gravity was low at 1.002.

### 2.4. Treatment

While in the emergency department, the patient received 2 liters of normal saline, along with supplementation of magnesium. Repeat labs four hours later showed an increase in sodium from 113 to 120 and continued to increase to 127 fourteen hours later. The patient denied neurologic symptoms. Given overcorrection of sodium within the first 14 hours with a concern of osmotic demyelination syndrome from overcorrection [1,2], nephrology was consulted urgently. They recommended lowering the sodium level by giving 500 cc of D5W over 2 hours, DDAVP 2mcg intravenously immediately, and then starting a D5W infusion at 100 cc/hour. The free water deficit formula based on weight was used to calculate the amount of free water needed to bring the patient back down to his target. After the above sodium lowering interventions, the repeat sodium level 20 hours after the initial presentation resulted at 119. The patient then received a small volume of normal saline in a controlled amount per nephrology's recommendation throughout his hospital stay, and his sodium was able to correct appropriately.

Given the patient's labs and history, he was diagnosed with hypo-osmolar hyponatremia from low solute intake, likely beer potomania given his history of binge drinking.

## 3. Discussion

To diagnose the underlying cause of hypo-osmolar hyponatremia, we require the help of urine electrolytes. We first look at urine osmolality, since urine osmolality is the nearest window to the body's ADH activity. ADH activation will increase the resorption of free water in the renal collecting duct, resulting in concentrated urine with high urine osmolality. Also, the opposite stays true; ADH suppression will result in low urine osmolality as a result [3].

In order to understand beer potomania and why it corrects rapidly with fluid, we first discuss the underlying pathophysiology. In the setting of hyponatremia, dilute urine with urine osmolality <100–200 mOsm/kg indicates that ADH is not activated. Several etiologies are known to cause hypo-osmolar hyponatremia without activation of ADH: tea-toast syndrome, primary polydipsia, and beer potomania. In our case, the patient had a urine osmolality of 94 and a history of binge drinking, pointing towards a diagnosis of beer potomania.

The cause of hyponatremia in beer drinking is related to the low solute in alcoholic beverages and protein insufficiency [4–6]. In a person with normal renal function, his or her kidney can maximally dilute urine to 50 mOsm/kg (or 50 mmol/L) [1, 6, 7] and maximally concentrate urine to 1200 mOsm/kg (or 1200 mmol/L). The amount of daily urine output will depend on a person's daily solute intake [2]; daily solute ingested will equal solute excreted under normal conditions. For example, if a 70 kg person ingests 10 mOsm/kg/day, his or her solute load will be 700 mOsm/day, which is excreted in the urine. A solute load of 700 mOsm/day divided by a maximally diluted urine concentration of 50 mmol/L will equal 14 L of urine output, and a solute load of 700 mOsm/day divided by maximally concentrated urine of 1200 mmol/L will equal around 0.6 L of urine output daily. In the same person, if he or she ingests 15 L of water, that extra 1 L will not be able to be excreted and sodium level will fall due to a dilutional effect [6]. In beer potomania patients, they usually have poor diet with low daily solute intake [8]. In our patient who has been drinking 12 cans of beer daily with poor diet due to jaw pain, we estimate that his daily solute intake is about 150 mOsm per day, which indicates that if his fluid intake is greater than 3 L per day, dilutional hyponatremia will result.

Beer potomania patients are highly prone to rapid sodium correction with intravenous fluid. Each liter of normal saline consists of 308 mOsm of solute, so the patient can now generate 6 L of urine from that one liter of fluid. This can cause rapid correction of sodium as he or she now has the capacity to produce six extra liters of urine, effectively lowering the blood sodium level as a result [2, 9]. As a result, it can be dangerous to give fluids without first delineating the underlying cause of hyponatremia, as in our case.

## Figures and Tables

**Figure 1:**
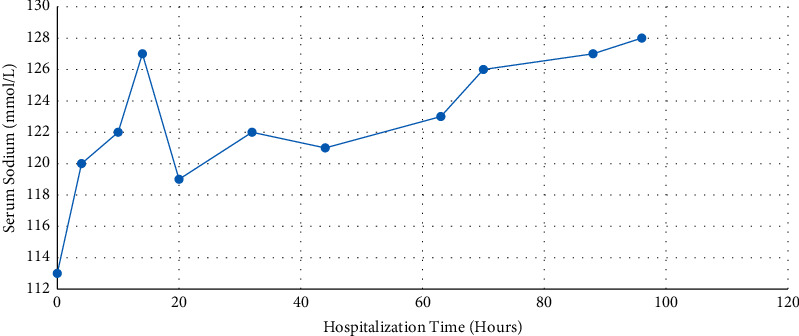
The Patient's sodium level throughout his hospital stay. He presented initially with a sodium level of 113 mmol/L at hour 0, then corrected to 127 mmol/L after receiving 2 liters of normal saline, which prompted a nephrology consult. The patient then received D5W and DDAVP, and sodium was able to lower to 119 mmol/L at hour 20.
